# Integrated Transcriptomic Analysis Identifies a Putative cfa-miR-10a–*ACTG1*/*SDC1* Network Associated with Extracellular Matrix and Cytoskeletal Remodeling in Canine Hepatocellular Carcinoma

**DOI:** 10.3390/cimb48080840

**Published:** 2026-08-19

**Authors:** Mohammad Arif, Most Shumi Akhter Shathi, Nobuhiro Nozaki, Naoki Miura

**Affiliations:** 1 Joint Graduate School of Veterinary Medicine, Kagoshima University, Kagoshima 890-8580, Japan; mdarif38515@bau.edu.bd (M.A.); shathi.vet@gmail.com (M.S.A.S.); non.21non.21no.z@gmail.com (N.N.); 2 Department of Microbiology and Hygiene, Bangladesh Agricultural University, Mymensingh 2202, Bangladesh; 3 Veterinary Teaching Hospital, Joint Faculty of Veterinary Medicine, Kagoshima University, Kagoshima 890-8580, Japan

**Keywords:** hepatocellular carcinoma, cfa-miR-10a, post-transcriptional regulation, extracellular matrix remodeling, cytoskeletal remodeling, *ACTG1*, *SDC1*, comparative oncology

## Abstract

A coordinated extracellular matrix (ECM) and cytoskeletal remodeling are critical drivers of hepatocellular carcinoma (HCC) progression, yet the upstream post-transcriptional mechanisms regulating these processes in spontaneous canine HCC remain poorly defined. Previously, we reported significantly downregulated miRNAs and enriched pathways from differentially expressed genes (DEGs) in canine HCC tissues. In the present study, we investigated putative post-transcriptional target interactions between the prioritized miRNAs and enriched ECM–cytoskeleton pathway-associated genes (*n* = 34). Integrative bioinformatic analysis prioritized *ACTG1* and *SDC1* as the key putative targets of cfa-miR-10a based on their concordant prediction by four target-prediction algorithms, high transcript abundance, and inverse correlations with cfa-miR-10a-5p. RNAhybrid and miRanda analyses supported favorable predicted interactions between cfa-miR-10a and the 3′UTRs of both transcripts, with RNAhybrid minimum free energy values ≤ −20 kcal/mol and miRanda scores ≥ 140. In the matched canine transcriptomic subset, cfa-miR-10a and its predicted targets showed inverse expression trends. Cross-species analysis using TCGA-LIHC datasets further demonstrated concordant dysregulation of *ACTG1*, *SDC1* and hsa-miR-10a-5p in human HCC, despite their weak or non-concordant correlation with hsa-miR-10a-5p. Collectively, these exploratory findings identify a candidate cfa-miR-10a–*ACTG1*/*SDC1* network associated with coordinated ECM and cytoskeletal transcriptomic alterations in canine HCC. While cross-species analysis supports the conservation of target gene dysregulation, the divergent miRNA-mRNA correlation patterns highlight potential species-specific post-transcriptional co-expression landscapes that warrant future functional validation.

## 1. Introduction

Hepatocellular carcinoma (HCC) is characterized by extensive remodeling of the tumor microenvironment (TME), particularly the extracellular matrix (ECM), which contributes to tumor progression and invasion [1,2]. In healthy liver tissue, the ECM maintains tissue architecture and cellular homeostasis; however, chronic inflammation and hypoxic stress promote pathological ECM remodeling, leading to matrix stiffening, altered cell adhesion, and enhanced epithelial–mesenchymal transition (EMT) [3,4].

The biological consequences of ECM remodeling are closely interconnected with mechanotransduction pathways that couple extracellular signaling to cytoskeletal reorganization during tumor progression [5]. Aberrant ECM composition and matrix stiffening signal through adhesion complexes to dynamically remodel the actin cytoskeleton, thereby promoting EMT, cancer cell invasion, and metastatic progression [6]. Among the cytoskeletal regulators involved in these processes, *ACTG1* (Actin Gamma 1) plays a central role in actin filament organization, mechanical adaptation, and tumor cell migration [7,8,9]. In HCC, *ACTG1* has additionally been associated with enhanced proliferation and resistance to apoptosis [10].

The transmembrane heparan sulfate proteoglycan *SDC1* (Syndecan-1) is another important mediator of ECM-associated signaling, functioning at the interface between extracellular ligands and intracellular structural systems such as the cytoskeleton. Through its interactions with integrins, growth factors, and cytoskeletal adaptor proteins, *SDC1* contributes to cell adhesion, stromal communication, and aggressive tumor phenotypes [11,12]. Given the coordinated interplay between ECM signaling and cytoskeletal remodeling in cancer progression, dysregulation of *ACTG1* and *SDC1* may contribute to ECM–cytoskeletal alterations in canine HCC. However, the upstream post-transcriptional mechanisms regulating *ACTG1*, *SDC1*, and other ECM–cytoskeleton-associated genes in canine HCC remain poorly understood.

MicroRNAs (miRNAs) are important post-transcriptional regulators of tumor-associated pathways, including ECM remodeling and epithelial plasticity. In our previous transcriptomic study, a set of 13 miRNAs, including cfa-miR-10a, were identified as significantly downregulated miRNAs in canine HCC tissues [13]. Based on our transcriptomic findings showing significant enrichment of ECM–cytoskeleton-associated pathways in canine HCC [14], we hypothesized that the downregulation of these miRNAs may be associated with dysregulation of ECM–cytoskeletal pathway-associated genes, thereby contributing to ECM and cytoskeletal remodeling in canine HCC. To investigate this potential association, we performed an integrative miRNA–mRNA interaction analysis focusing on the ECM–cytoskeleton-associated genes identified from the significantly enriched pathways. In other oncogenic contexts, miR-10a has been implicated in regulating cell motility and EMT by modulating cytoskeletal and adhesion-associated molecules, notably by directly targeting *ACTG1* in colorectal cancer [15] and *SDC1* in epithelial malignancies [16]. However, the potential involvement of these downregulated miRNAs in regulating ECM–cytoskeleton-associated genes in hepatocellular carcinoma, particularly in the context of ECM and cytoskeletal remodeling, remains largely unexplored. Therefore, in the present study, we systematically evaluated the potential regulatory relationships between the selected downregulated miRNAs and 34 ECM–cytoskeleton-associated genes using multiple target-prediction algorithms, transcript abundance, and miRNA–mRNA expression correlations. This pathway-focused analysis enabled prioritization of candidate miRNA–mRNA interactions and identified a putative cfa-miR-10a–*ACTG1*/*SDC1* relationship in canine HCC. Given the inherent limitations of our small transcriptomic cohort, this work aims to provide a prioritized computational and transcriptomic framework for understanding miRNA-associated ECM–cytoskeletal regulation and guiding future cross-species functional validation.

## 2. Materials and Methods

### 2.1. Bioinformatic Prediction and Prioritization of miRNA–mRNA Interactions

Thirteen miRNAs previously identified as significantly downregulated in canine HCC [13] were selected for network analysis. These comprised eight canine-annotated miRNAs and five miRNAs annotated using human reference sequences in the original dataset. For the latter, canine homologous miRNAs were identified based on sequence homology and canine miRNA annotation. Where a homologous canine miRNA was already represented among the eight canine-annotated miRNAs, the existing canine entry was retained to avoid duplication. Where no distinct canine homolog was identified, the human-annotated miRNA was excluded from the network analysis. This resulted in a final set of nine unique miRNAs, comprising eight canine miRNAs and one human-reference miRNA with a corresponding canine homolog, which were used for construction of the comprehensive miRNA–mRNA regulatory network.

A curated set of 34 ECM–cytoskeleton-associated genes was selected based on the ECM and cytoskeleton-related pathways previously identified as among the most significantly enriched pathways in canine HCC in our earlier study [14]. Putative targets of the nine selected downregulated miRNAs were then evaluated specifically within this predefined 34-gene set. Target prediction was performed using *Canis lupus familiaris*-specific TargetScan and miRDB, applying cumulative weighted context++ scores ≤ −0.4 and miRDB prediction scores ≥ 80, respectively. In parallel, potential miRNA–mRNA interactions were assessed using miRanda (v3.3a) and RNAhybrid (v2.1.2) against the corresponding 3′UTR sequences of the 34 candidate genes, applying stringent criteria of MFE ≤ −20 kcal/mol for RNAhybrid and MFE ≤ −15 kcal/mol with a miRanda score ≥ 140 for miRanda. Putative miRNA–gene interactions were defined as those involving one of the predefined 34 ECM–cytoskeleton-associated candidate genes and supported by at least two of the four prediction algorithms. The resulting interactions were subsequently integrated with transcript abundance and miRNA–mRNA expression correlations for network construction and target prioritization.

### 2.2. Protein–Protein Interaction (PPI) Network Construction and Hub Gene Identification

To elucidate the functional interactions and structural connectivity among ECM–cytoskeleton-associated genes (*n* = 34), a PPI network was constructed. Interacting protein pairs were predicted using the Search Tool for Retrieval of Interacting Genes/Proteins (STRING v12.0) database, with a medium confidence threshold of ≥0.4. The resulting interactome was imported into Cytoscape (v3.10.4) for comprehensive network visualization and topological analysis. To isolate the most structurally critical nodes driving the network, the cytoHubba plug-in was utilized. The top 10 foundational hub genes were computed and ranked using the Maximal Clique Centrality (MCC) algorithm.

### 2.3. Expression Profiling and Correlation Analysis

We leveraged matched small RNA-seq and mRNA-seq transcriptomic datasets previously generated by our laboratory [13,14] for expression profiling of cfa-miR-10a and its target genes. The sequencing sub-cohort comprised independent, unpaired liver tissue samples (total *n* = 6), including histopathologically confirmed canine HCC tissues (*n* = 3) resected from client-owned patients at the Kagoshima University Veterinary Teaching Hospital and normal liver tissues (*n* = 3) obtained from adult laboratory Beagle dogs. The normal liver tissues were remnant tissues from a separate study and were provided by the Drug Safety Research Laboratories of Shin Nippon Biomedical Laboratories, Ltd. (SNBL DSR, Kagoshima, Japan). No animals were used or euthanized specifically for the present study to obtain normal liver tissues. Comprehensive animal metadata are provided in Table S1. Total RNA was isolated using the mirVana™ miRNA Isolation Kit (Thermo Fisher Scientific, Waltham, MA, USA) according to the manufacturer’s instructions, as previously detailed [13,14]. For each individual animal tissue, small RNA and mRNA sequencing were executed from the same total RNA extraction. To assess potential inverse regulatory relationships, normalized expression values generated following read mapping and transcript quantification using CLC Genomics Workbench (v24.0) (QIAGEN, Hilden, Germany) were used to calculate Spearman’s correlation coefficients (*r*) between each of the nine selected miRNAs and the 34 ECM–cytoskeleton-associated candidate genes across the matched canine transcriptomic cohort.

### 2.4. Evaluation of Cross-Species Conservation in Human HCC

To assess cross-species conservation, human orthologs were evaluated using the ENCORI (StarBase v2.0), UALCAN, and GEPIA2 portals. Expression profiles and miRNA–target correlations were analyzed in the TCGA-LIHC (Liver Hepatocellular Carcinoma) cohort. Prognostic value was determined using the ENCORI and UALCAN databases, with the log-rank test used to compare high- and low-expression groups.

### 2.5. Statistical Analysis and Visualization

All statistical calculations and correlation matrices were executed using GraphPad Prism (v8.0). In our canine transcriptomic datasets, the expression levels of cfa-miR-10a and its target genes were compared between normal liver and HCC groups using the Mann–Whitney U test. The linear relationships between miRNA and target gene expression profiles were evaluated via Spearman’s correlation analysis (*r*). Given the small sample size (*n* = 3 HCC vs. *n* = 3 normal), Spearman’s correlation coefficients (*r*) and associated *p*-values are interpreted purely as exploratory trend indicators rather than stable population metrics. For all statistical tests, significance thresholds were strictly set at a two-tailed *p* < 0.05. Data visualizations, including a volcano plot, a protein–protein interaction (PPI) network, a hub gene network, expression plots, and Spearman correlation plots, were generated using ggplot2 (v4.0.3), Cytoscape (v3.10.4), and GraphPad Prism (v8.0).

## 3. Results

### 3.1. Identification of a Core ECM–Cytoskeletal Remodeling Signature in Canine HCC

**Figure 1:**
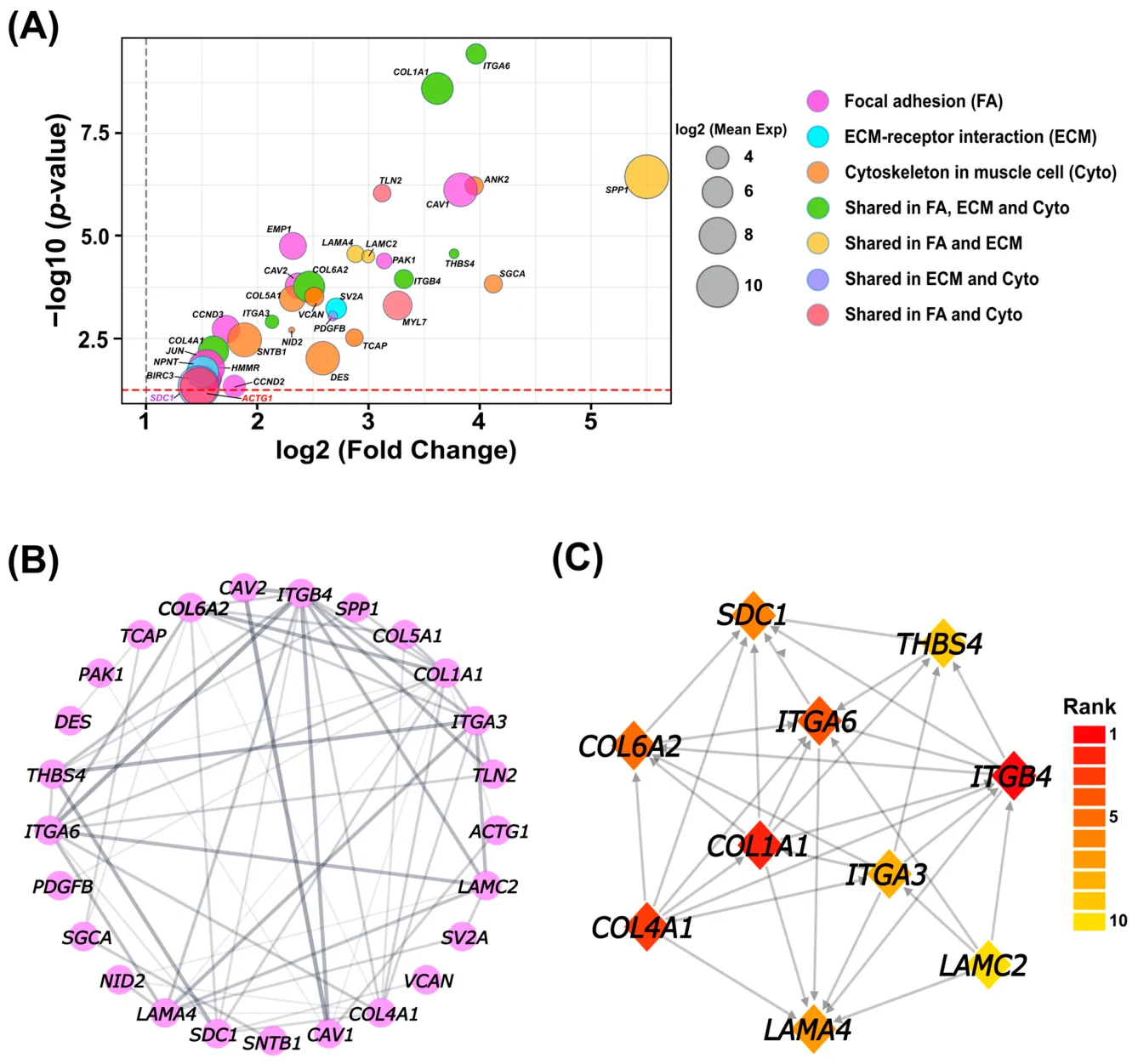
Identification and prioritization of an ECM–cytoskeleton-associated gene signature in canine HCC. (**A**) Volcano plot illustrating the distribution of 34 differentially expressed genes (DEGs) across three prioritized ECM–cytoskeleton-associated pathways (Focal Adhesion, ECM–Receptor Interaction, and Cytoskeleton in Muscle Cells). Plot points are color-coded by pathway annotation, with bubble dimensions reflecting log_2_ maximum group mean expression levels. (**B**) Protein–protein interaction (PPI) network illustrating interactions among all ECM–cytoskeleton-associated DEGs. Genes lacking protein–protein interactions were not included in the PPI network visualization. (**C**) Subnetwork highlighting the top 10 hub genes ranked using the Maximal Clique Centrality (MCC) algorithm.

Building on our previous transcriptomic analysis of canine HCC [14], we focused on three ECM–cytoskeleton-associated pathways among the top five significantly enriched pathways: Focal Adhesion, ECM–Receptor Interaction, and Cytoskeleton in Muscle Cells (Table S2). Intersection analysis identified seven shared genes (*ITGA3*, *ITGB4*, *THBS4*, *COL1A1*, *COL4A1*, *COL6A2*, and *ITGA6*) across all three pathways (Table S3). Differential expression metrics for the 34 ECM–cytoskeleton-associated genes were obtained from our previously published RNA-seq analysis [14]. All 34 ECM–cytoskeleton-associated candidate genes were significantly upregulated in the complete canine HCC transcriptomic datasets (log_2_ fold change > 1 and *p* < 0.05; Table S4). Among these, *SPP1* exhibited the greatest magnitude of upregulation (log_2_ fold change > 5.5) in the dataset, occupying a distinct position on the volcano plot (Figure 1A). Furthermore, *SPP1*, *ACTG1*, and *SDC1* displayed the largest bubble dimensions on the volcano plot, highlighting their exceptionally high baseline expression profiles (log_2_ maximum group mean expression ≈ 10) relative to the other candidate genes, while *COL1A1* and *ITGA6* demonstrated the highest statistical significance (−log_10_ *p*-value > 8) combined with substantial expression magnitudes (Figure 1A). Protein–protein interaction (PPI) analysis demonstrated extensive connectivity among the 34 ECM–cytoskeleton-associated genes (Figure 1B). Subsequent cytoHubba analysis prioritized a cluster of hub nodes, including the collagen family (*COL1A1*, *COL4A1*, *COL6A2*), integrin subunits (*ITGB4*, *ITGA6*, *ITGA3*), ECM glycoproteins (*LAMA4*, *LAMC2*, *THBS4*), and the proteoglycan *SDC1* (Figure 1C).

### 3.2. Integrative miRNA–Target Interaction Network Prioritizes ACTG1 and SDC1 as Putative cfa-miR-10a Target Hubs

**Figure 2:**
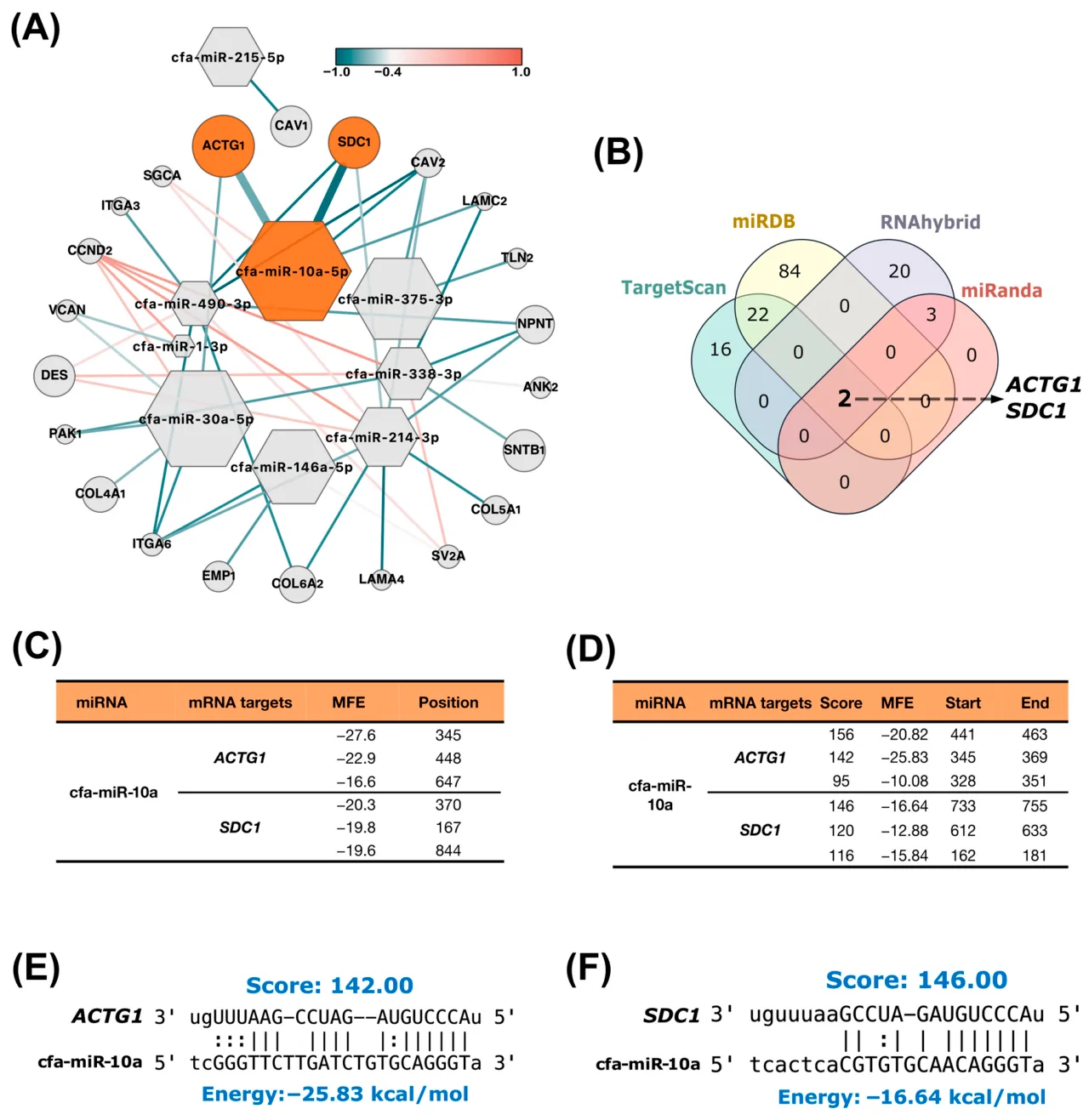
Comprehensive miRNA–target interaction network of ECM–cytoskeletal candidates, as well as structural modeling, prioritizes *ACTG1* and *SDC1* as potential cfa-miR-10a target hubs in canine HCC. (**A**) Integrated miRNA–target gene interaction network illustrating predicted regulatory relationships across the 34 ECM–cytoskeletal candidate genes. Node shapes distinguish miRNAs (hexagons) from target mRNAs (circles). Node size reflects relative transcript abundance in canine HCC tissues. Orange highlighting indicates the prioritized microRNA (cfa-miR-10a-5p) and its top predicted target gene nodes (*ACTG1* and *SDC1*), while non-highlighted nodes are shown in grey. Edge thickness indicates algorithmic prediction confidence (thick edges = consensus across 4 prediction databases; thin edges = prediction by 2 thermodynamic algorithms). Edge color gradient highlights Spearman rank correlation coefficients (*r*), ranging from strong inverse correlations (dark teal, *r* ≤ −0.7) to positive correlations (coral). (**B**) Venn diagram showing the consensus overlap among TargetScan, miRDB, RNAhybrid, and miRanda for cfa-miR-10a target candidate identification. (**C**,**D**) Predicted binding site parameters, minimum free energy (MFE) values, and alignment scores for cfa-miR-10a interactions with *ACTG1* and *SDC1* transcripts generated via RNAhybrid (**C**) and miRanda (**D**). (**E**,**F**) Detailed sequence alignment models showing seed-region complementarity between cfa-miR-10a and the 3′UTRs of *ACTG1* (**E**) and *SDC1* (**F**).

To systematically investigate whether the downregulated miRNAs identified in canine HCC are potentially connected to the ECM–cytoskeletal remodeling signature, we constructed an integrated miRNA–mRNA regulatory network encompassing 9 prioritized downregulated miRNAs and the 34 ECM–cytoskeletal genes by integrating TargetScan, miRDB, RNAhybrid, and miRanda predictions with transcript abundance and Spearman correlation analysis using the matched canine transcriptomic cohorts (Figure 2A; Tables S4–S9). Among the 34 pathway-associated candidate genes, *ACTG1* and *SDC1* uniquely met all prioritization criteria, including prediction by all four algorithms (Figure 2B, Tables S6 and S9), relatively high transcript abundance in HCC tissues (based on transcriptomic data; Table S4), and computationally predicted strong inverse correlations with cfa-miR-10a-5p (*r* = −0.71 and −0.94, respectively; Table S9). Notably, *ACTG1* and *SDC1* ranked second and third, respectively, in transcript abundance among the 34 candidate genes (Table S4). These findings placed *ACTG1* and *SDC1* as the top putative targets of cfa-miR-10a within the ECM–cytoskeletal gene set.

In silico thermodynamic analyses using RNAhybrid and miRanda demonstrated favorable predicted interactions between cfa-miR-10a and both target transcripts. For *ACTG1*, both algorithms identified a candidate binding site at nucleotide position 345, with a minimum free energy (MFE) of −27.6 kcal/mol predicted by RNAhybrid, and a miRanda alignment score of 142 (Figure 2C,D; Tables S7 and S8). Similarly, *SDC1* showed stable predicted interactions at positions 370 (MFE: −20.3 kcal/mol) and 733 (Score: 146) (Figure 2C,D; Tables S7 and S8). Sequence alignment analysis further demonstrated strong seed-region complementarity between cfa-miR-10a and the predicted target sites (Figure 2E,F). Collectively, these findings support a putative cfa-miR-10a–*ACTG1*/*SDC1* candidate network, suggesting a hypothetical framework where miRNA deficiency may align with the observed overexpression of these ECM–cytoskeletal transcripts in canine HCC.

### 3.3. Expression Profiling Supports Inverse Relationships Between cfa-miR-10a and ACTG1/SDC1

**Figure 3:**
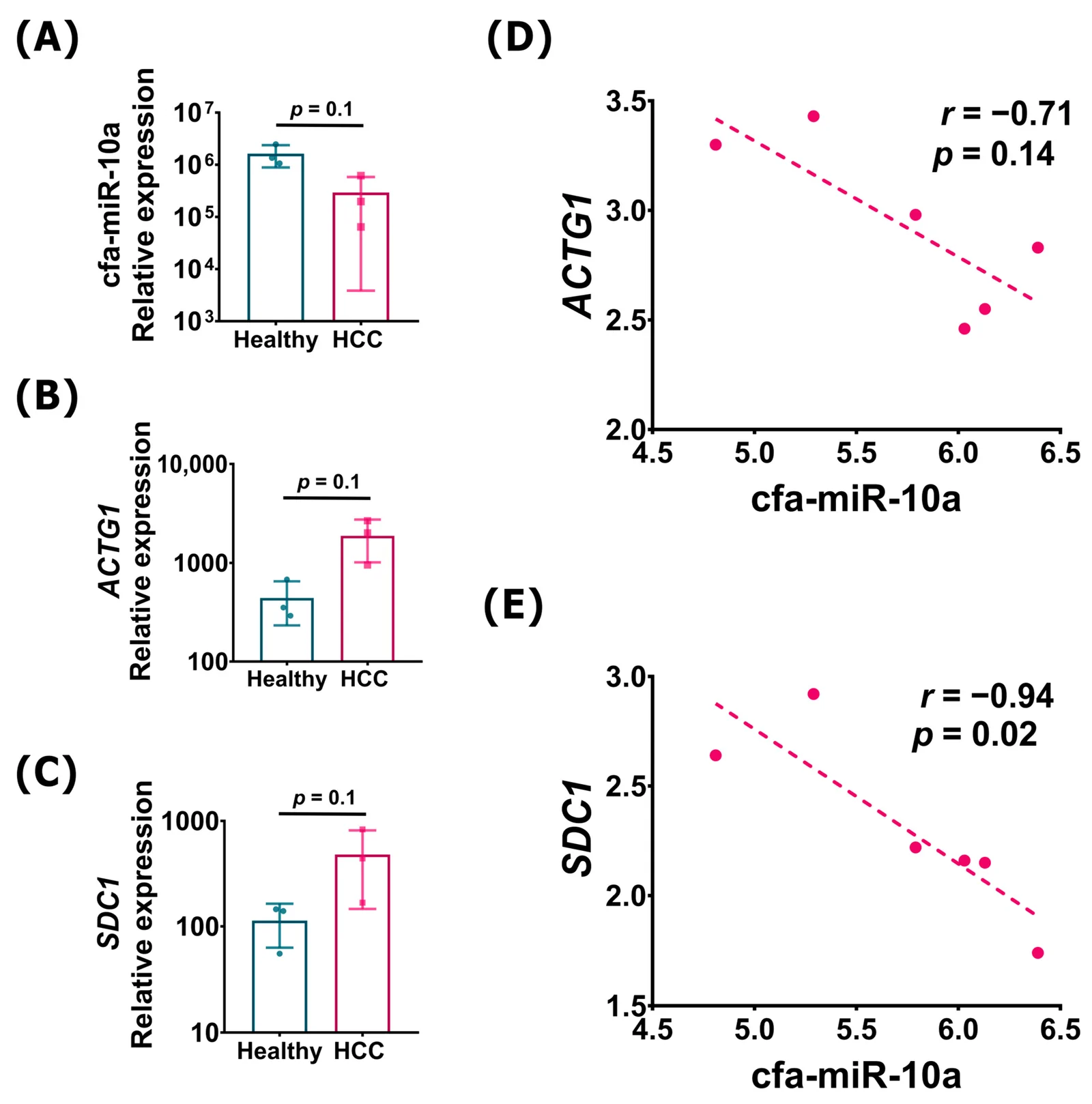
Transcriptomic expression profiles and inverse associations between cfa-miR-10a and *ACTG1*/*SDC1* in canine HCC. (**A**–**C**) Normalized transcript expression levels of cfa-miR-10a (**A**), *ACTG1* (**B**), and *SDC1* (**C**) derived from transcriptomic datasets of healthy liver tissues (*n* = 3) and canine HCC tissues (*n* = 3) for exploratory expression and correlation analysis. Data points represent individual biological samples, with bars indicating mean ± standard deviation (SD). Exact *p*-values from Mann–Whitney U tests are shown within each panel. These comparisons are based on the limited six-sample subset and are distinct from the genome-wide differential-expression analysis performed on the complete transcriptomic dataset. (**D**,**E**) Spearman correlation analysis showing inverse associations between cfa-miR-10a and *ACTG1* (**D**) or *SDC1* (**E**) expression profiles. Spearman correlation coefficients (*r*) and two-tailed *p*-values are indicated within each panel.

To integrate the in silico target predictions with transcriptomic expression profiles, we analyzed the relationship between cfa-miR-10a and its predicted targets, *ACTG1* and *SDC1*. Aligning with our previous RT-qPCR validation of cfa-miR-10a downregulation in an independent cohort [13], the current exploratory transcriptomic dataset mirrored this downward expression pattern in canine HCC tissues (Figure 3A), while *ACTG1* and *SDC1* showed reciprocal upward expression trends (Figure 3B,C). These expression patterns were consistent with the differential-expression results obtained from the complete transcriptomic dataset (Table S4).

Spearman rank correlation analysis was performed to evaluate the inverse associations between cfa-miR-10a and both target genes within this limited cohort (*n* = 6). *SDC1* demonstrated a significant negative correlation with cfa-miR-10a (*r* = −0.94, *p* = 0.02; Figure 3E). Conversely, the inverse correlation for *ACTG1* failed to achieve statistical significance (*r* = −0.71, *p* = 0.14; Figure 3D). Given the small sample size, these correlation estimates should be interpreted with caution, as they are exploratory and intended to generate hypotheses rather than establish definitive biological relationships.

### 3.4. Comparative Evaluation of the miR-10a–ACTG1/SDC1 Relationship in Human HCC

**Figure 4:**
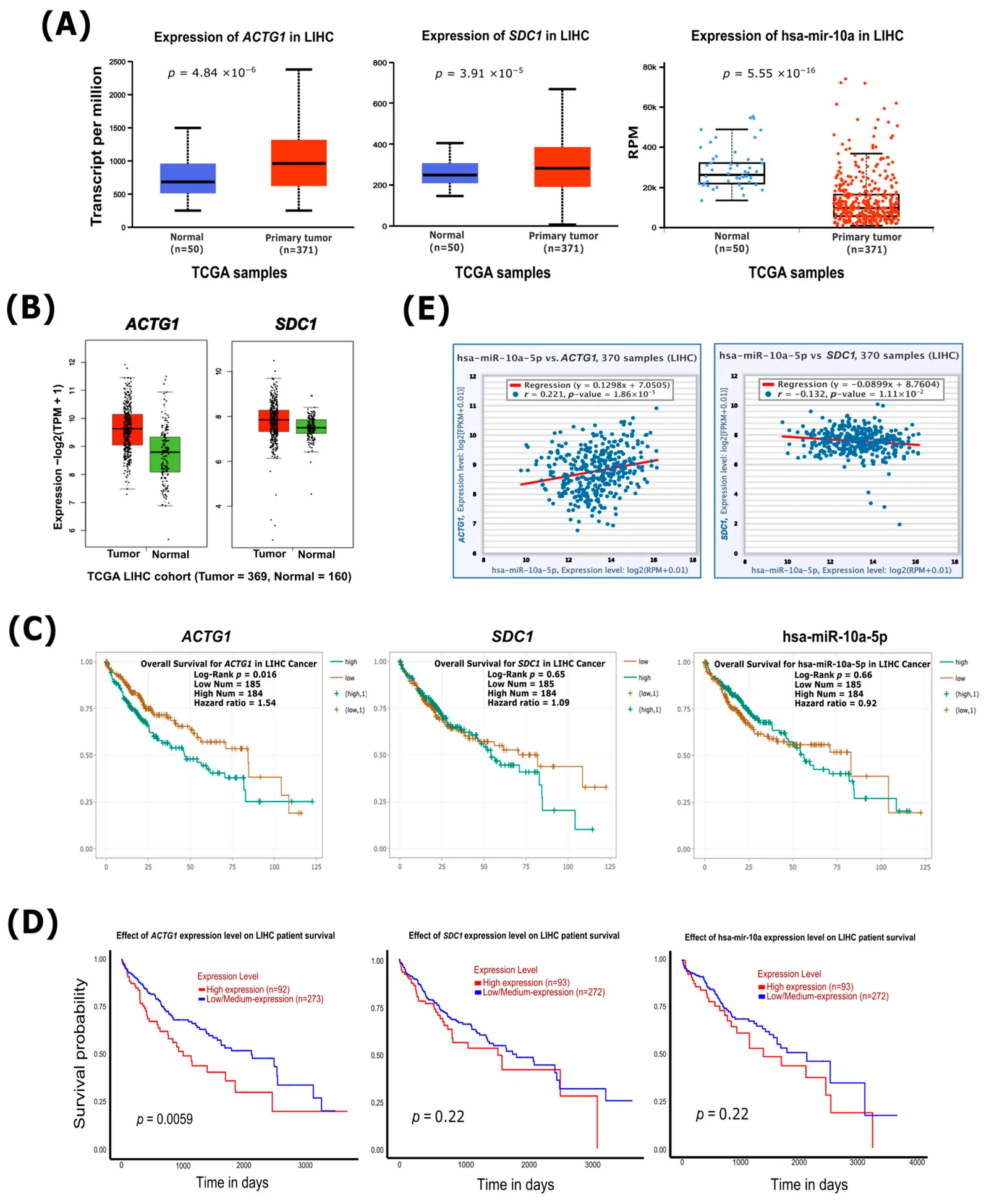
Transcriptional profiling, survival analysis, and hsa-miR-10a-5p (the human homolog of cfa-miR-10a) correlation of *ACTG1* and *SDC1* in human HCC (TCGA-LIHC). (**A**) Expression levels of *ACTG1*, *SDC1*, and hsa-miR-10a between primary liver hepatocellular carcinoma (LIHC) tumor tissues (*n* = 371) and adjacent normal liver tissues (*n* = 50) retrieved from the UALCAN database. (**B**) mRNA expression validation of *ACTG1* and *SDC1* in TCGA-LIHC tumor (*n* = 369) versus normal (*n* = 160) samples using GEPIA2. (**C**,**D**) Kaplan–Meier overall survival analysis for *ACTG1*, *SDC1*, and hsa-miR-10a in LIHC patients derived from ENCORI (**C**) and UALCAN (**D**). (**E**) Correlation analysis between hsa-miR-10a-5p and target gene expression (*ACTG1* and *SDC1*) using ENCORI. Spearman correlation coefficients (*r*) and corresponding *p*-values are indicated within each panel.

Building upon our recent finding that hsa-miR-10a-5p exhibits altered expression in human HCC (TCGA-LIHC) [13], we evaluated the integrated expression landscape of hsa-miR-10a-5p alongside its putative targets, *ACTG1* and *SDC1*, in human HCC. While *ACTG1* and *SDC1* showed marked upregulation in human HCC tissues relative to normal liver controls, hsa-miR-10a-5p exhibited significant downregulation, reflecting an opposing expression trend consistent with our observations in canine HCC (Figure 4A,B). Survival analysis using ENCORI showed that high *ACTG1* expression was significantly associated with poorer overall survival (*p* = 0.016), whereas *SDC1* (*p* = 0.65) and hsa-miR-10a-5p (*p* = 0.66) were not significantly associated with overall survival (Figure 4C). Similarly, UALCAN analysis demonstrated a significant association between high *ACTG1* expression and reduced patient survival (*p* = 0.0059), while neither *SDC1* nor hsa-miR-10a-5p showed a significant survival association (*p* = 0.22 for both; Figure 4D).

We next evaluated the relationship between hsa-miR-10a-5p and the corresponding putative target genes in the human cohort. In contrast to the canine dataset, correlation analysis revealed weak or negligible associations in human HCC (*SDC1*: *r* = −0.132; *ACTG1*: *r* = 0.221; Figure 4E). Although hsa-miR-10a-5p and its targets showed conserved differential expression, the human bulk-transcriptome correlations did not recapitulate the inverse relationships observed in canine HCC. These findings suggest that the relationship between hsa-miR-10a-5p and its putative targets may be influenced by context- or species-specific regulatory mechanisms.

### 3.5. Candidate Framework of Predicted cfa-miR-10a-Associated ECM–Cytoskeletal Interactions

**Figure 5:**
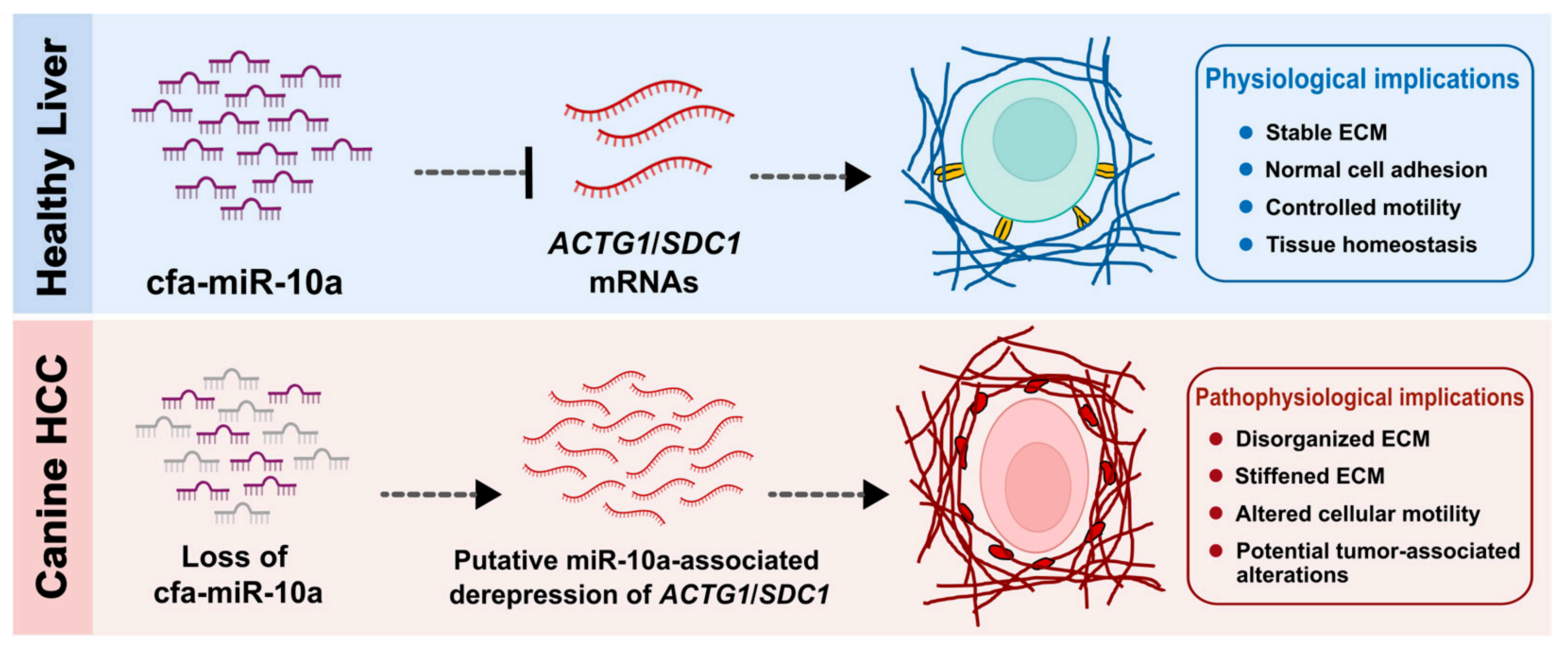
Conceptual framework of putative cfa-miR-10a-associated transcriptomic alterations linked to ECM and cytoskeletal remodeling in canine hepatocellular carcinoma. Under normal liver conditions (blue background/panel), baseline cfa-miR-10a levels are computationally predicted to target *ACTG1* and *SDC1* expression (dashed line with blunt inhibition bar), potentially correlating with (dashed arrow) the maintenance of normal ECM and cytoskeletal transcript homeostasis. In canine HCC (red background/panel), reduced cfa-miR-10a expression may contribute to increased *ACTG1* and *SDC1* expression through putative loss of post-transcriptional regulation (dashed arrows). Dysregulation of these ECM–cytoskeleton-associated genes may be associated with altered ECM organization, cytoskeletal remodeling, and enhanced tumor-associated cellular dynamics.

In summary, we present a conceptual model (Figure 5) outlining a predicted transcriptomic network wherein reduced cfa-miR-10a expression in canine HCC aligns with increased *ACTG1* and *SDC1* abundance. Under normal liver conditions, baseline cfa-miR-10a levels are computationally predicted to target *ACTG1* and *SDC1*, potentially correlating with the maintenance of normal ECM and cytoskeletal transcript homeostasis. In the malignant state, the depletion of this miRNA coincides with the marked upregulation of these targets, highlighting a putative axis for coordinated ECM–cytoskeletal transcriptomic remodeling in canine HCC. Collectively, this prioritized candidate framework supports a potential link between cfa-miR-10a expression patterns and microenvironmental/cytoskeletal structural dynamics during canine hepatocarcinogenesis, providing a clear foundation for future cross-species functional validation.

## 4. Discussion

The orchestration of the tumor microenvironment is increasingly recognized as a non-cell-autonomous process regulated by microRNA-mediated networks [17]. In the present study, we identified a putative cfa-miR-10a–*ACTG1*/*SDC1* transcriptomic network in canine HCC, wherein computational modeling and exploratory expression profiling suggest an inverse expression association between downregulated cfa-miR-10a and the upregulated transcripts *ACTG1* and *SDC1*. Collectively, these exploratory findings highlight a putative transcriptomic link between ECM alterations and cytoskeletal remodeling in canine hepatocarcinogenesis, potentially involving shared post-transcriptional regulatory mechanisms that warrant future functional validation.

To map microenvironmental disruption in canine HCC, our network screening delineated a core ECM interactome enriched for integrins, collagens (*COL1A1*, *COL4A1*, *COL6A2*), and the proteoglycan *SDC1*. Notably, *SPP1* emerged as a prominent transcriptomic feature with the highest fold change and baseline expression. These ECM-associated transcriptomic alterations underscore extensive ECM remodeling in canine hepatocarcinogenesis [4]. Among the identified hub genes, *SDC1* was particularly noteworthy, not only for its identification within the hub network but also for its remarkably high transcript abundance, ranking third among all ECM–cytoskeleton-associated genes in the canine HCC transcriptomic dataset (Table S4). This dual characteristic supports the potential role of *SDC1* as an important ECM-associated regulatory node.

The integrated analysis of the 34 ECM–cytoskeleton-associated genes identified *ACTG1* and *SDC1* as the highest-priority putative targets of cfa-miR-10a. Both genes showed support across four independent target-prediction algorithms, relatively high transcript abundance in canine HCC tissues, and inverse correlations with cfa-miR-10a-5p, providing convergent computational evidence supporting their prioritization as putative miRNA-regulated transcripts. In silico modeling further indicated favorable interactions between cfa-miR-10a and the 3′UTRs of both transcripts. However, the expression correlation analysis should be interpreted cautiously because it was based on only six matched tissue samples (three HCC and three normal tissues), which substantially limits statistical power. Although *SDC1* demonstrated a significant negative correlation (*r* = −0.94, *p* = 0.02), the correlation for *ACTG1* failed to achieve statistical significance (*r* = −0.71, *p* = 0.14). The lack of statistical significance for *ACTG1* may therefore reflect the limited sample size and consequent low power to detect an association, rather than the absence of an inverse expression relationship. Nevertheless, these correlations remain exploratory and cannot establish direct miRNA-mediated regulation. Experimental validation in a larger cohort and functional assays will be necessary to determine whether cfa-miR-10a directly regulates *ACTG1* and *SDC1* in canine HCC.

Consequently, while these targets collectively participate in ECM-associated signaling and cytoskeletal remodeling, any hypothesis suggesting that cfa-miR-10a depletion directly drives their reciprocal expression or facilitates coordinated microenvironmental adaptations remains strictly hypothetical. While requiring confirmation in larger cohorts, such concurrent alterations could potentially reflect the structural adaptations and altered mechanotransduction dynamics triggered by a remodeling ECM, which are known to facilitate tumor cellular migration and invasion [4].

Dysregulation of these ECM–cytoskeletal regulators is consistent with the established human HCC literature. *ACTG1* overexpression has been associated with enhanced proliferation, chemoresistance, evasion of apoptosis, and EMT-mediated motility in HCC [7,10,18]. Similarly, *SDC1* dysregulation and its proteolytic shedding into the tumor microenvironment have been linked to EMT progression, matrix remodeling, and aggressive tumor behavior [19,20,21]. Within our exploratory dataset, the parallel dysregulation of *ACTG1* and *SDC1*, together with their predicted interaction with a common miRNA, provides a candidate framework for investigating potential links between *SDC1*-associated ECM signaling and *ACTG1*-associated cytoskeletal organization in canine hepatocarcinogenesis. This hypothetically implicates mechanotransduction pathways that dynamically connect extracellular matrix alterations to intracellular structural remodeling in HCC, providing a speculative baseline that requires future functional validation to confirm this candidate relationship and to determine whether modulation of these components (e.g., using miRNA mimics) alters their expression or relevant cellular phenotypes.

Although *ACTG1* and *SDC1* overexpression was observed in human HCC datasets, a significant association with reduced overall survival was identified only for *ACTG1*. Moreover, the weak and non-concordant correlations with hsa-miR-10a-5p, including a positive correlation with *ACTG1*, indicate that the proposed inverse regulatory relationship observed in canines was not recapitulated at the bulk-transcriptome level in human HCC. This divergence may partly reflect the greater molecular and etiological heterogeneity of human HCC, which encompasses diverse disease backgrounds, including viral hepatitis and alcohol-associated liver disease [22]. In addition, the overexpression of *ACTG1* and *SDC1*, observed in TCGA databases, is likely regulated by multiple miRNAs and transcriptional programs in human HCC, which may obscure individual miRNA–mRNA relationships in bulk transcriptomic data [23,24]. Differences in tumor composition, stromal content, tumor purity, and species-specific 3′UTR architecture may further contribute to the observed divergence. In contrast, cfa-miR-10a may display a more prominent inverse correlation profile in the canine context. The absence of a conserved inverse correlation does not exclude biological relevance but suggests context- and species-dependent regulation, highlighting the potential utility of the canine model for isolating specific candidate oncogenic drivers that are masked in human populations [25]. Nevertheless, the consistent upregulation of *ACTG1* and *SDC1* across canine and human HCC, together with the significant prognostic association observed for *ACTG1*, supports further investigation of this candidate network in larger, clinically stratified human cohorts to determine whether subtype-specific or context-dependent hsa-miR-10a-5p-mediated regulation exists.

While our integrative approach identifies a compelling candidate network, several important limitations should be acknowledged. First, our transcriptomic cohort (*n* = 3 HCC vs. *n* = 3 normal liver) was exploratory in nature. Although matched miRNA and mRNA profiles generated from the same tissue specimens provide internal consistency for integrative analyses, the limited sample size reduces statistical power and limits the biological generalizability and stability of correlation estimates. Accordingly, the identified cfa-miR-10a–*ACTG1*/*SDC1* candidate network should be regarded as hypothesis-generating rather than definitive. Second, in the absence of experimental validation, including dual-luciferase reporter assays, Ago2-RIP assays, miRNA gain- and loss-of-function experiments, or protein-level analyses such as Western blotting, a direct causal interaction between cfa-miR-10a and *ACTG1* or *SDC1* cannot be established. Thus, the proposed candidate network remains computationally inferred and descriptive. Finally, the lack of an inverse miRNA–mRNA relationship in the human TCGA-LIHC dataset suggests that this regulatory interaction may be species-specific or restricted to particular molecular or clinical contexts. Alternatively, *ACTG1* and *SDC1* expressions in human HCC may be regulated through a more complex and redundant miRNA regulatory network that differs from the canine system.

## 5. Conclusions

In the present study, we conducted an exploratory integrative analysis to prioritize putative miRNA–mRNA regulatory relationships associated with ECM–cytoskeletal remodeling in canine HCC. The analysis identified a putative cfa-miR-10a–*ACTG1*/*SDC1* relationship supported by multiple computational and transcriptomic approaches. However, these findings remain hypothesis-generating and do not establish direct miRNA-mediated regulation or functional involvement in ECM–cytoskeletal remodeling. Given the limited transcriptomic cohort and lack of experimental validation, further studies using larger cohorts and functional assays are required to validate these candidate interactions and determine their biological significance.

## Data Availability

The canine HCC RNA-Seq datasets utilized in this study were previously generated by our group and are publicly available in the NCBI BioProject database under accession number PRJNA1293103 (https://www.ncbi.nlm.nih.gov/bioproject/?term=PRJNA1293103; accessed on 18 July 2025). Other data are available within the article and the supporting materials.

## Supplementary Materials

The following supporting information can be downloaded at https://www.mdpi.com/article/10.3390/cimb48080840/s1.

## Abbreviations

The following abbreviations are used in this manuscript:

3′UTR	3′ Untranslated Region
*ACTG1*	Actin Gamma 1
Ago2-RIP	Argonaute 2-RNA Binding Protein Immunoprecipitation
DEG	Differentially Expressed Gene
ECM	Extracellular Matrix
EMT	Epithelial–Mesenchymal Transition
ENCORI	Encyclopedia of RNA Interactomes
GEPIA2	Gene Expression Profiling Interactive Analysis 2
HCC	Hepatocellular carcinoma
KEGG	Kyoto Encyclopedia of Genes and Genomes
LIHC	Liver Hepatocellular Carcinoma
MCC	Maximal Clique Centrality
MFE	Minimum Free Energy
miRNA/miR	MicroRNA
NCBI	National Center for Biotechnology Information
NGS	Next-Generation Sequencing
PPI	Protein–Protein Interaction
RT-qPCR	Reverse Transcription-Quantitative Polymerase Chain Reaction
*SDC1*	Syndecan-1
*SPP1*	Secreted Phosphoprotein 1
STRING	Search Tool for Retrieval of Interacting Genes/Proteins
TCGA	The Cancer Genome Atlas
TME	Tumor Microenvironment
